# New Therapeutic Scenarios in the Context of Adjuvant Treatment for HR+/HER2−Breast Cancer: The Possible Role of Ribociclib in Treatment Algorithms for Stage II and III

**DOI:** 10.3390/curroncol32040192

**Published:** 2025-03-25

**Authors:** Nicola Battelli, Carmela Mocerino, Michele Montedoro, Mirco Pistelli, Ilaria Portarena, Mario Rosanova, Tina Sidoni, Patrizia Vici

**Affiliations:** 1 Oncologia, Ospedale di Macerata, 62100 Macerata, Italy; nicola.battelli@sanita.marche.it; 2 Unità di Oncologia, A.O.R.N. Cardarelli, 80131 Napoli, Italy; carmela.mocerino@aocardarelli.it; 3 Ospedale di Città di Castello, 06012 Città di Castello, Italy; michele.montedoro@gmail.com; 4 Dipartimento di Oncologia, Ospedali Riuniti di Ancona, 60126 Ancona, Italy; pistelli.mirco@gmail.com; 5 Dipartimento di Oncologia, Policlinico Tor Vergata, 00133 Roma, Italy; ilaria_portarena@hotmail.com; 6 Unità di Oncologia, Ospedale del Mare, 80147 Napoli, Italy; rosanovamario@hotmail.com; 7 Ospedale San Salvatore, 67100 l’Aquila, Italy; tina_sidoni@yahoo.it; 8 UOSD Sperimentazioni di Fase IV, IRCCS Istituto Nazionale Tumori Regina Elena, 00144 Roma, Italy

**Keywords:** breast cancer, adjuvant therapy, ribociclib, stage II, stage III, adherence

## Abstract

Early breast cancer (EBC) treatment has evolved from radical surgery to a multidisciplinary approach, integrating radiotherapy, chemotherapy, targeted therapy, and hormone therapy with surgery to ensure the best possible outcome. Despite these advancements, hormone receptor-positive (HR+)/Human Epidermal Growth Factor Receptor 2-Negative (HER2−) EBC still faces high recurrence rates after endocrine therapy. A panel of oncologists from Central-Southern Italy discussed the profile of ribociclib as an adjuvant therapy, based on the results of the NATALEE study, focusing on efficacy, safety, patient profiles, and regional challenges in treatment access. The experts identified ribociclib as suitable adjuvant treatment for stage II and III HR+/HER2− EBC patients, including those without lymph node involvement but with biologically aggressive disease. In their view, ribociclib could be an interesting option for patients not eligible for chemotherapy due to contraindications. Key challenges in translating the evidence on ribociclib in EBC into clinical practice include treatment duration, patient follow-up, and adverse events management. Strategies to address these challenges range from telemedicine and support from local clinics to tailored communication to improve adherence. Ribociclib is expected to significantly impact adjuvant treatment for HR+/HER2− EBC by addressing broader patient needs and potentially improving long-term outcomes through enhanced adherence and personalized management strategies.

## 1. Introduction

Breast cancer treatment has evolved significantly since the 1970s, shifting from demolitive surgery to an integrated, multidisciplinary approach that combines various therapies for optimal outcomes. Although surgery remains the primary treatment for localized breast cancers, the therapeutic approach now includes radiotherapy, chemotherapy, targeted therapy, and hormone therapy [1]. The majority of breast cancer cases are diagnosed at an early stage (EBC), with stage I being the most common and stage II diagnoses occurring around three times more frequently than stage III diagnoses [2]. Despite recent progress, recurrences remain a concern, with the first years being most at risk for triple-negative and Human Epidermal Growth Factor Receptor 2 (HER2) positive disease, while long term recurrences are a significant issue for hormone receptor positive (HR+) HER2 negative (HER2−) disease. HR+/HER2− EBC is typically treated with upfront surgery, followed by radiotherapy and chemotherapy when indicated, and then 5 to 10 years of endocrine therapy [3]. However, the risk of relapse is still high after these treatments, with the tumor potentially returning even 5–20 years after the initial diagnosis [4,5]. In fact, with a 20-year follow-up, about 30% of patients with stage II disease and up to 50% of those with stage III experience a metastatic recurrence [6]. Moreover, about 50% of relapses occur more than 5 years after surgery [7].

Endocrine therapy-based treatment is the first choice for HR+ HER2− metastatic breast cancer, according to national and international guidelines [8,9]. This preference is due to its effectiveness and the lower incidence of severe side effects compared to cytotoxic chemotherapy, which remains indicated as a first line treatment only in situations of imminent organ failure [8,9]. Combining endocrine therapy with cyclin-dependent kinase 4 and 6 (CDK4/6) inhibitors has demonstrated superior efficacy to endocrine therapy alone in several studies [10,11,12,13]. The shift of CDK4/6 inhibitors from advanced to early settings has been explored and is now gaining increasing attention [14]. Abemaciclib was the first CDK4/6 inhibitor to show a statistically significant reduction in invasive disease-free survival (iDFS) in a selected population in the adjuvant setting. In the monarchE study, the addition of abemaciclib to standard endocrine therapy for patients with node-positive, high-risk EBC resulted in a 29% reduction in the risk of iDFS compared to endocrine therapy alone [15,16]. The benefits of abemaciclib extend beyond the two-year treatment period, suggesting that it provides durable protection against recurrence, even after treatment has ended [17]. Nevertheless, the monarchE study included only a small portion of the real-world HR+/HER2− EBC population typically encountered in everyday clinical practice, leaving a broader group still at risk of relapse with an unmet need, including a subset of those without lymph node involvement.

In 2023, data from the NATALEE study showed that ribociclib, when used as adjuvant therapy in a broad population of HR+/HER2− EBC patients, including those with stage II and III disease without nodal involvement but at risk of recurrence, significantly improved iDFS, with HR 0.75, 95% confidence interval (CI) 0.62–0.91; *p* = 0.003 [18,19].

The NATALEE study also demonstrated an improvement in distant relapse-free survival, a secondary endpoint, while overall survival data are still immature at this early stage of follow-up [19]. These results address the unmet need for effective adjuvant treatments across different stages of EBC, irrespective of nodal involvement.

These new adjuvant therapies present clinical challenges in managing breast cancer patients. According to U.S. data, about 50% of patients with EBC are non-adherent to endocrine therapy, and up to 73% discontinue therapy before the recommended timeframe [20,21,22]. A systematic review confirmed that adherence is a challenge in the adjuvant context, with age, cancer side effects, and poor quality of life (QoL) being key influencing factors [23]. Low adherence significantly impacts survival, leading to an increased risk of disease recurrence or death.

Here we report the outcomes of a discussion among a panel of Italian experts on the integration of ribociclib in the adjuvant treatment for HR+/HER2− stage II and III EBC, with specific attention to the peculiarities of different regional situations in Italy and the existing challenges. The aim is to characterize the potentially eligible profile of early-stage HR+/HER2− breast cancer populations, evaluate adjuvant therapy management, and assess adverse events and their impact on QoL, to improve the decision-making process for clinical patient management.

## 2. Materials and Methods

A panel of nationally recognized oncologists, selected by Novartis for their extensive experience in breast cancer treatment, gathered for a round-table meeting in March 2024 in Rome, organized by Novartis. They represented diverse clinical settings, from university hospitals to community practices, to capture a wide array of perspectives across neighboring regions in Central-Southern Italy. The discussion was guided by a moderator, who facilitated the exchange of insights and ensured a structured, in-depth analysis of key topics. The meeting included a full overview of the clinical studies discussing the use of CDK 4/6i as adjuvant therapy, followed by an in-depth analysis of potential future changes in the therapeutic landscape within the clinical practice of the Italian centers represented by the panelists. The panel discussed the patient profile characteristics that influence the choice of ribociclib as adjuvant therapy, as well as the regional factors that could be a barrier or facilitate treatment access.

The discussion was mainly informed by data from the NATALEE study, recent literature evidence [16,17,19,24], and the experts’ clinical experience.

## 3. Results

### 3.1. Drivers for Treatment Choice: Patient Profile and Risk Characterization

The board agreed that, based on their experience and the Italian regulatory context, adjuvant treatment with ribociclib would be particularly promising for patients in stage II and other high-risk stages where there is a clear indication of risk but where abemaciclib is not indicated. The expert panel considers this therapy as addressing an unmet need in current clinical practice, covering a broad population with stage II and III disease whose risk is high, even with negative lymph nodes.

In finer detail, patients with positive lymph nodes who do not meet the criteria for abemaciclib, as well as those with negative lymph nodes but biologically aggressive disease, such as a high expression of Ki67, are considered among the most interesting candidates for ribociclib adjuvant therapy. There is also great interest in ribociclib as a viable option for those patients who should receive adjuvant chemotherapy but cannot because of contraindications.

**Figure 1:**
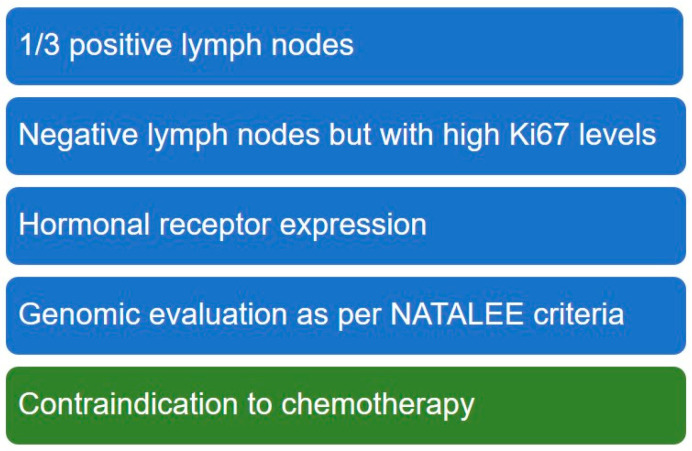
Possible factors defining the population where ribociclib adjuvant treatment could have the greater impact.

Besides lymph node involvement and biological factors evaluated through Ki67 levels, another factor to be considered in therapy choice is the level of expression of hormone receptor. Genomic evaluation is perceived as a minor influence on the choice and should be included only to better characterize G2 tumor, as done in the inclusion criteria of the NATALEE trial (Figure 1).

### 3.2. Challenges and Strategies in Managing Adjuvant Therapy with Ribociclib

The expert panel highlighted that introducing adjuvant ribociclib therapy in stage II and III EBC may present some challenges. The NATALEE trial introduced a lower dose of ribociclib (400 mg instead of 600 mg used in the metastatic setting) and an extended treatment duration in comparison to other CDK 4/6 inhibitors used in the adjuvant setting (3 years instead of 2 years) (Table 1) [17,19,24]. The duration of treatment together with the reduced dose of 400 mg of ribociclib have been implemented in the NATALEE study to optimize efficacy while improving tolerability, considering the totality of evidence from preclinical and clinical research. Although longer treatment duration could potentially impact overall QoL, data from NATALEE confirm that patient QoL is not impacted by the treatment, with similar functioning and global health status in the two arms of the study [25]. The three-year treatment duration [19] was discussed, particularly in terms of the burden on patients (number of visits, perception of “ongoing illness” by the patients). While concerned for this aspect, the panel does not see the three-year duration as a limiting factor, as long as this is the best therapeutic choice for the patients. The duration of ribociclib treatment appears manageable, especially considering the potential benefits for the patient.

The main difficulties are related to patient follow-up. Indeed, the first few months of therapy are critical, as adverse events and compliance issues are more likely to occur [26]. This is particularly true for individuals who have already received chemotherapy. Dose adjustment is often required to achieve a balance between efficacy and tolerability, and this implies frequent contact with patients. Clinicians pointed out a common shortage of personnel and facilities in their areas, with consequent issues in appropriate patient management. In addition, the limited availability of Breast Units and dedicated clinics, which are not widespread in the area of interest of the meeting, can make it difficult for patients to receive optimal care and support. These organizational issues might represent critical red flags in managing adjuvant therapy with ribociclib, as the potential target population eligible for treatment would be larger than the one eligible for abemaciclib in this setting. Nevertheless, this increased burden should not impact the decision to provide beneficial treatment.

### 3.3. Implication of Adherence and QoL in Therapy Management

Adherence in an adjuvant setting is challenging, and international data show that a great proportion of patients do not follow their prescribed therapy [23]. Despite this, the experts report that the Italian scenario is not characterized by such a high rate of non-adherence to adjuvant therapies. Although challenging, in the experience of panelists there is overall consistency in daily drug administration, without a high proportion of non-adherence. Factors influencing adherence to adjuvant therapy include patient age and polypharmacy. One of the panelists shared her hospital’s experience with a survey administered to the patients, which enabled the identification of the most common barriers to adherence, confirming that the addition of an oral medication may be hard to manage for those already taking multiple drugs.

Clinicians emphasized that in the metastatic setting, abemaciclib-associated side effects, particularly diarrhea and gastrointestinal issues, have a more pronounced impact on patients’ QoL compared to those of ribociclib. Ribociclib’s side effects, such as neutropenia, hypertransaminasemia, and QTcF interval prolongation, are typically subclinical and do not have an immediate or noticeable effect on QoL.

However, they also acknowledge that tolerability is subjective and adverse events may be perceived differently in the adjuvant setting compared to the metastatic setting, potentially leading to treatment discontinuation. In the metastatic setting, adverse events are usually tolerated as patients are well aware of the importance of the therapy and less likely to discontinue the treatment.

In this light, a better communication of the risk of recurrence and the benefits of adjuvant therapy by both clinicians and surgeons is crucial to improve adherence. To enhance communication to patients, the board suggests involving surgeons, to effectively explain post-surgery outcomes to patients and highlight the value of adjuvant treatment. This approach would help to facilitate patients’ understanding of the significance of adjuvant therapy.

## 4. Discussion

The integration of CDK4/6 inhibitors represents a significant advancement in the treatment of HR+ HER2− breast cancer. CDK4/6 inhibitors have been shown to be highly effective in treating HR+ HER2− advanced or metastatic breast cancer [11,12,13]. In HR+/HER2− EBC, adjuvant endocrine therapy is the standard care; however, the risk of recurrence persists, particularly with longer follow-up. Clinical studies have reported a sustained risk of recurrence over the long term, with rates of up to 52% at 20 years, correlating to nodal involvement at the time of diagnosis [6]. Real-world data from across the US support the high risk of recurrence, reaching 40.5% at 10 years for patients with stage II disease and 62.9% for stage III, emphasizing the necessity for improved therapeutic options for EBC patients [27].

The new frontier in the treatment of HR+ HER2− breast cancer is represented by the integration of CDK4/6 inhibitors in the adjuvant setting. CDK4/6 inhibitors revolutionized the treatment of metastatic disease [11,12,13] and are now showing promises in the early setting [15,18,19]. Abemaciclib is currently used in adjuvant clinical practice, while ribociclib recently received approval for the adjuvant setting by FDA and EMA highlighting the urgency of the topic [28,29] (Table 1).

**Table 1:** A summary of adjuvant CDK4/6 inhibitor trials.

Trial	CDK 4/6 Inhibitor	Patient Population	Treatment Duration	Primary Endpoint	*p* Value	EMA Approval
monarchE [17]	Abemaciclib	HR+/HER2− EBC: Cohort 1: ≥4 ALN or 1–3 ALN + additional risk factors Cohort 2: 1–3 ALN + Ki-67 ≥ 20%	2 years	4-year-iDFS HR (95% CI): 0.66 (0.58–0.76)	*p* < 0.0001	Approved [30]
NATALEE [19]	Ribociclib	HR+/HER2− EBC (Stage II and III)	3 years	3-year-iDFS HR (95% CI): 0.75 (0.62–0.91)	*p* = 0.003	Approved [29]
PALLAS [24]	Palbociclib	HR+/HER2− EBC (Stage II and III invasive BC)	2 years	4-years-iDFS HR (95% CI): 0.96 (0.81–1.14)	*p* = 0.65	Not approved

To discuss the future therapeutic landscape of the adjuvant setting, with a special focus on the Italian scenario, a panel of Italian clinicians, experts in the clinical management of HR+ HER2− breast cancer, gathered for a roundtable meeting.

The experts agreed that the introduction of ribociclib as an additional option in the adjuvant setting for EBC would be an opportunity, as it would allow for the treatment of a broader patient population. This includes those with stage II and III HR+/HER2− EBC without lymph node involvement, that were not included in the monarchE trial, who are at risk of disease recurrence up to decades after the initial diagnosis [31,32], especially if they also present an aggressive biology. The stage and the high level of Ki67 are defined as the major drivers for treatment eligibility.

The subset of patients without lymph node involvement represented 28.1% of the whole NATALEE population at diagnosis, and among this subgroup, the HR for iDFS favored the ribociclib arm (0.63, 95% CI: 0.34–1.16) [19]. These findings were further supported by data presented at ESMO 2024, reinforcing the observed improvement both in the overall population and in the N0 subgroup [33].

Ribociclib could represent an option for patients who are not eligible for abemaciclib therapy, which is indicated for patients with the involvement of four or more lymph nodes, or with one to three lymph nodes providing they have additional risk factors [15]. Ribociclib would represent the only option for those patients who do not meet the abemaciclib criteria (including those without nodal involvement). The use of ribociclib would therefore address a major current unmet need in clinical practice by being offered to a broader population. Moreover, in the NATALEE study, 12% of the included population had not received previous chemotherapy [19]. In this light, ribociclib would provide reassurance to oncologists in cases where the pathological history of patients prevents them from receiving chemotherapy. For these patients, treatment with CDK4/6i represents an opportunity for the intensification of adjuvant therapy, offering a chance to upscale treatment.

The increased number of patients potentially eligible to ribociclib therapy may pose an issue in costs [34] and patient management. The financial impact of treatment will need to be carefully considered, including an evaluation of the burden of metastatic recurrence, which not only entails higher long-term healthcare costs but also substantially affects patients’ QoL and survival outcomes. Real-world cost-effectiveness analyses considering country-specific healthcare systems and indirect costs will help to assess the economic impact of ribociclib in the adjuvant setting. Future analyses incorporating long-term follow-up data will provide complete information on the overall value of this therapeutic approach. Considering patients’ management, the expert panel expect that practical strategies can be implemented to ensure the best treatment for all patients, as challenges accompanying new therapy should not influence the treatment decision. Although some patients might feel discouraged by the prospect of a three-year therapy, the experts highlighted that better communication to empower patients would support adherence. To improve patient management, considering the potential increased burden on specialized centers, suggested strategies are the use of telemedicine, support from local clinics, and extended prescription schedules.

While treatment discontinuation was similar in the monarchE and NATALEE studies (18.5% vs. 19.5%, respectively) [17,35], the distinct toxicity profiles of the treatments should be carefully considered when selecting the most appropriate therapy for each patient, as distinct patient populations may benefit from different treatments in the adjuvant setting. Diarrhea (83.6%), neutropenia (45.9%), and fatigue (40.8%) were the most common all-grade AEs reported with abemaciclib treatment, while neutropenia (19.6%) was the most common grade ≥ 3 AE, followed by leukopenia (11.4%) and diarrhea (7.8%) [17]. Neutropenia (62.5%), arthralgia (37.3%), and nausea (23.3%) were all-grade AEs reported for ribociclib. The most common grade ≥ 3 AE was neutropenia (44.3%). Grade ≥ 3 liver-related AEs occurred in 8.6% of patients. Liver-related AEs resolved within approximately 3 weeks following protocol-guided dose adjustments [35]. Moreover, each patient should be assessed individually, considering comorbidities, age, and concomitant medications, to determine the most suitable drug for each case.

Effective communication is strongly believed to help patients understand the positive role of adjuvant therapy as an essential and non-optional treatment [36]. Improved patient understanding of the benefits of the treatment can also enhance adherence. Although adherence is often more complex in non-metastatic patients who may underestimate the general risk of disease relapse, good communication between patients and their oncologists/surgeons can increase patient awareness and allow clinicians to gather more information about patients’ needs and feelings [37,38]. Some centers have already adopted strategies to better involve patients in the therapeutic process, such as administering dedicated questionnaires. Tailored communication that addresses the needs of patients can improve the quality of care and, eventually, therapy outcomes [39]. According to the experts’ experience, responses to questionnaires have provided valuable real-world information about perceived adverse events, such as the recurrence of arthralgia, allowing clinicians to better prepare patients for these possible outcomes.

Other tools, including telemedicine, should be explored, and smartphone application could be considered [40] to improve patient assistance and reduce the burden of several patient–clinician interactions.

Adverse events are a major challenge in maintaining adherence to therapy. Aromatase inhibitors are generally not well tolerated in pre-menopausal patients [41]. Adverse events such as insomnia, anxiety, fatigue, and bone pain should also be considered for CDK4/6 inhibitors because they negatively affect patients’ QoL [42]. Nevertheless, ribociclib treatment was well-tolerated [19]. In the NATALEE study, adverse events were mainly registered at the early phases of treatment [19], with no major influence on QoL. Adverse events with the use of abemaciclib have been primarily reported in the first 2–3 months of treatment, and therefore this phase of therapy requires intensive follow-up of patients to maintain adherence [23]. Closely monitoring patients during the first months of therapy is advisable for prompt intervention in case of need, for instance, considering dose reduction. This intervention can mitigate AEs while maintaining treatment efficacy, as shown with abemaciclib [15]. Should the therapy be well-tolerated, the experts suggest a long-term prescription to reduce the burden of frequent visits to the center for care.

The specialized centers in the area are generally well-organized, and despite the low numbers of breast units, they could be better prepared to provide multidisciplinary support to patients by setting up a network with territorial clinics.

Besides the current evidence presented by the NATALEE study, the experts expressed interest in further data on a possible switch from abemaciclib to ribociclib in case of toxicity. Future studies and real-world evidence may provide further insights into this aspect. Moreover, there is a need for deeper efficacy data on the population with *BRCA1/2* gene mutation in the adjuvant setting. While poly(adenosine diphosphate-ribose) polymerase inhibitor showed improved OS in the adjuvant setting for EBC patients with germline variants *BRCA1* or *BRCA2* [43], the response to CDK4/6i in patients with *BRCA1/2* mutations is an area of ongoing research.

In conclusion, ribociclib would be a welcome addition to adjuvant therapy, addressing the need to treat a large population at risk of recurrence that is currently excluded from therapy with abemaciclib. A careful evaluation of patient characteristics, including age, comorbidities, ongoing medications, is essential to define the profile for which the treatment would be most beneficial in the adjuvant setting. Improving communication skills should be a priority for healthcare providers in oncology, to ensure patient empowerment, informed participation in therapeutic choices, and better adherence to treatment.

## Data Availability

The original contributions presented in this study are included in the article. Further inquiries can be directed to the corresponding author.

## Abbreviations

The following abbreviations are used in this manuscript:

AE	Adverse Events
AI	Aromatase Inhibitor
ALN	Axillary Lymph Node
CDK	Cyclin-Dependent Kinase
EBC	Early breast cancer
HER2−	Human Epidermal Growth Factor Receptor 2-Negative
HR+	Hormone Receptor-positive
iDFS	invasive Disease-Free Survival
PFS	Progression-Free Survival
QoL	Quality of Life
QTcF	QT interval corrected for heart rate using Fridericia’s formula
